# Biologics During Pregnancy and Breastfeeding Among Women With Rheumatic Diseases: Safety Clinical Evidence on the Road

**DOI:** 10.3389/fphar.2021.621247

**Published:** 2021-02-11

**Authors:** Asmaa Beltagy, Azin Aghamajidi, Laura Trespidi, Wally Ossola, Pier Luigi Meroni

**Affiliations:** ^1^ Istituto Auxologico Italiano, IRCCS, Immunorheumatology Research Laboratory, Milan, Italy; ^2^ Rheumatology and Clinical Immunology Department, Faculty of Medicine, Alexandria University, Alexandria, Egypt; ^3^ Department of Immunology, School of Medicine, Iran University of Medical Sciences, Tehran, Iran; ^4^ Department of Obstetrics and Gynaecology, Fondazione Ca Granda, Ospedale Maggiore Policlinico, Milan, Italy

**Keywords:** biologics, conception, pregnancy, breastfeeding, rheumatic diseases

## Abstract

Females are generally more affected by autoimmune diseases, a fact that underlines the relationship with pregnancy and the safety of anti-rheumatic drugs in pregnancy and lactation. Biologic therapies are increasingly prescribed to treat and maintain remission in a significant number of systemic autoimmune rheumatic diseases. The experience with the use of biologics during gestation is extremely lacking because of the observational nature of the available studies and the difficulty in designing proper clinical trials in pregnancy. Among the studied biologics, more information was published on TNFα inhibitors and, in particular, on their potential passage through the placenta and impact on the fetus. Currently, a fragment of anti-TNFα monoclonal IgG, certolizumab pegol, is considered safe with almost no placental transfer. Subsequent observations are suggesting a comparable safety for the soluble TNFα receptor etanercept. Another biologic, eculizumab, the anti-C5a antibody used to treat complement-mediated microangiopathies, is also considered safe due to the unique engineered IgG2/4κ formulation that limits its passage through the placental barrier. Still, long-term data about children born to women treated with biologics in pregnancy are not attainable. Data on breastfeeding are currently available for several biologics. This article reviews the literature available about which drugs are considered safe during pregnancy and lactation, which are not, and on future prospects.

## Introduction

Autoimmune diseases affect around 3%–5% of the population. The prevalence is high for some diseases like rheumatoid arthritis (RA) representing ≈0.5%–1% and low for other diseases like systemic sclerosis (≈0.04%) (Jacobson et al., 1997; Cooper and Stroehla, 2003; Gabriel and Michaud, 2009; Schirmer et al., 2012). Systemic autoimmune rheumatic diseases (SARD) are generally more common in women during reproductive age, with female to male ratio up to 13:1 in diseases like systemic lupus erythematosus (SLE) and Sjogren syndrome, making antirheumatic drug exposure during pregnancy and lactation a frequent issue (Jacobson et al., 1997; Petri, 2002; Fava and Petri, 2019).

Pregnancy and SARD are reciprocally related; a flare in disease activity can occur in pregnancy and disease activity negatively affects pregnancy course and outcome (Andreoli et al., 2019; Giles et al., 2019). Therefore, it is crucial to reach and maintain total or near total remission before and during pregnancy for good pregnancy outcomes. Conventional drugs sometimes do not to achieve this therapeutic target, and safe drug choices are limited in pregnancy (Kuriya et al., 2011; Götestam Skorpen et al., 2016; Ngian et al., 2016).

The management of SARD has changed significantly with the revolutionary advent of biological disease-modifying antirheumatic drugs (bDMARD). Prescription of biologics to millions of patients with SARD has surged remarkably over the last two decades. Common SARD treated with bDMARDs include RA, SLE, spondyloarthritidis (SpA), juvenile idiopathic arthritis (JIA), and autoinflammatory syndromes. Upgrading treatment to biologics led to better control of disease activity that was considered resistant, improved quality of life, and prevented long-term functional disabilities of many patients with SARD (Shadick et al., 2019).

It is not uncommon to encounter a woman who desires a pregnancy or has unplanned pregnancy while on treatment with biologic therapies. Hence, several queries arise about issues related to the gestational safety and efficacy of this particular treatment. The overall experience with the use of biologics and the quality of evidence is not as strong as it should be since most of the available studies are observational with limited capability to conduct experimental trials in pregnancy (Sammaritano et al., 2020). More knowledge about long-term outcomes of children born to mothers treated with biologics in pregnancy is still needed, and it is expected to grow with ongoing studies. In 2016, two sets of recommendations were issued guiding the use of conventional or biological DMARDs in pregnant females with rheumatic diseases with not much discrepancy between them except for addressing paternal use and specifying the timing for stoppage of some drugs before conception by the British Society of Rheumatology and British Health Professionals in Rheumatology (BSR-BHPR) guidelines (Flint et al., 2016; Götestam Skorpen et al., 2016; Wu and Ying, 2019). Updated recommendations were published early 2020 with more consistency and evidence for safety (Sammaritano et al., 2020).

In this article, we review the literature available concerning what is considered safe during pregnancy and lactation, what is not, and the future prospects.

## General Concepts

### General Structure of Biologics

Currently, biologics employed for the treatment of SARD are either immunoglobulin G (IgG) full monoclonal antibodies (mAbs) or antibody fragments. IgG are generally characterized by high antigen affinity with less off-target activity (Strohl and Strohl, 2012). IgG1 is the most experimented and clinically used IgG subclass in biologics development, while very few types are constructed of other IgG isotypes (IgG2 and 4) (Ryman and Meibohm, 2017). The switch from chimeric to humanized to fully human antibodies improved the safety and tolerability of mAbs. Direct targets of mAbs are mostly inflammatory mediators such as tumor necrosis factor alpha (TNFα), interleukin 17 (IL-17), IL-6, IL-1, B-cell activating factor (BAFF), and less commonly cell markers (e.g., CD20) and cytokine receptors (e.g., IL-6R).

Although the functional part of the antibody is the antigen binding site, keeping the Fc portion intact preserves the molecule’s long half-life. On the other hand, using antibody fragments enables better distribution and tissue penetration but with a shorter half-life and less functional affinity to the target molecule. As an alternative, adding an attachment to the antibody fragment such as polyethylene glycol (PEG) moiety prevents proteolysis, decreases renal clearance, and maintains a reasonable drug half-life (Schmid and Neri, 2019).

### Maternal-Fetal Transport of Immunoglobulins

IgG and low levels of IgA are the only antibodies transferred from the mother to the fetus. Early in pregnancy, insignificant amounts of IgG are slowly transported by passive diffusion. Only 5–10% of the maternal level was measured at the end of the first trimester in blood samples obtained by cordocentesis (Jauniaux et al., 1995; Malek et al., 1996). A significant spike in maternal IgG transfer to the fetus starts at weeks 17–22 and increases thereafter resulting in higher, or at least similar, IgG levels in cord blood than maternal circulation at term, providing the newborn with a temporary passive immunity (Jauniaux et al., 1995; Malek et al., 1996; Saji et al., 1999; Simister, 2003; Lozano et al., 2018). The transfer is supported by placental structures such as neonatal Fc receptors (FcRn) as well as other placental Fcγ receptors (FcγR) (Wood et al., 1982).

**FIGURE 1:**
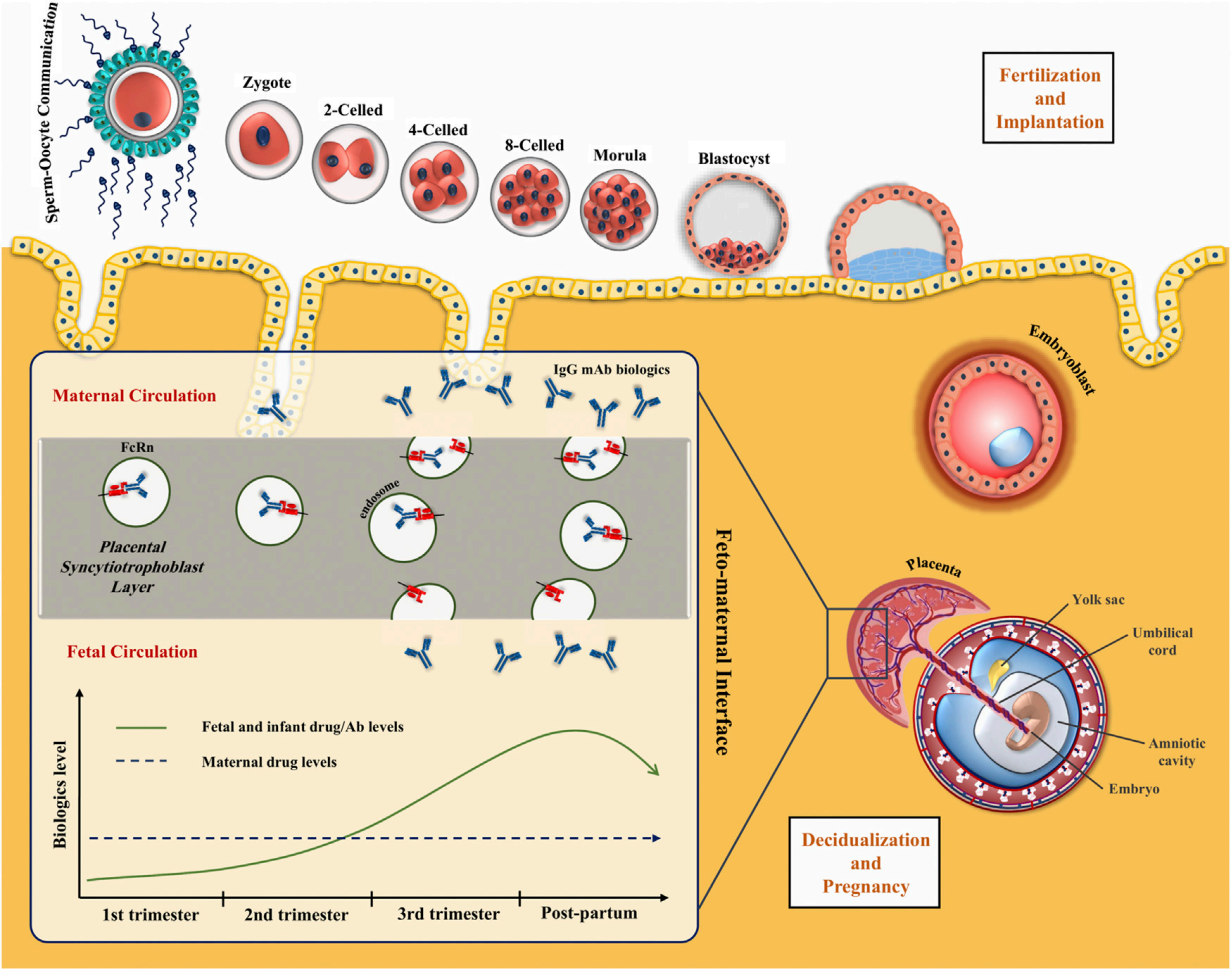
Placental transfer of IgG mAbs. IgG is the only antibody class that crosses the placenta through the syncytiotrophoblast layer. FcRn internalizes the maternal IgG in the endosome and releases it on the other side of the syncytiotrophoblast layer. IgG transmission significantly increases during the third trimester of pregnancy.

At the maternal-fetal interface, FcRn are expressed on the placenta together with the three types of FcγR. However, it has been demonstrated that FcRn promotes the most IgG transport via a process of binding at the syncytiotrophoblast layer, endocytosis, and release into the fetal circulation (Figure 1) (Firan et al., 2001). IgG1 are preferentially transported, followed by IgG4, IgG3, and IgG2 (Leach et al., 1996; Simister et al., 1996; Kane and Acquah, 2009; Lozano et al., 2018). On the other side, FcRn on maternal endothelial cells and liver are responsible for maintaining IgG half-life including therapeutic mAbs via endosomal recycling and re-expression to the cell surface and unbinding (Junghans and Anderson, 1996; Pyzik et al., 2019).

Based on these concepts, whole mAbs with the Fc domains, particularly IgG1, will be transferred at high levels to the fetus through placental FcRn. Antibody formulations lacking the Fc portion or less readily transferable IgG subclass like IgG2, will keep the fetus relatively safe from the effects of the biologic therapy. The main concern about the transfer of bDMARD to the fetus is their theoretical potential to disrupt children`s immunity and response to infections and vaccinations.

## Biological Drugs Used in Rheumatology

### TNFα Inhibitors

Currently, five anti-TNFα agents are being used in patients with various SARD, notably RA, SpA, and JIA among others (Monaco et al., 2015). They are the most studied in relation to pregnancy. Treatment with anti-TNFα drugs is usually accompanied by co-prescription of methotrexate (MTX), which should be stopped 1–3 months prior to attempting conception or when pregnancy is confirmed. Interestingly, anti-TNFα drugs were recently proposed as a potential treatment for refractory obstetric antiphospholipid syndrome (APS) (Alijotas-Reig et al., 2019).

Most of the data available about TNFα inhibitors, in particular infliximab, use in pregnancy are from studies on inflammatory bowel disease (IBD) patients. Observations from animal studies simulated to an extent what is being observed in humans. Despite transfer of anti-TNFα mAbs during organogenesis, no changes in fetal immune responses were reported. Safety with exposure throughout pregnancy and lactation was also ensured despite persistence of the drug up to 6 months in the blood of the exposed offspring (Treacy, 2000; Martin et al., 2007; Martin et al., 2008; Arsenescu et al., 2011).

#### Outcomes of Human Pregnancies with TNFα Inhibitors

The first retrospective analysis, from Centocor’s infliximab safety database, included 96 pregnancies in women diagnosed mostly with RA and Crohn`s disease (CD) (94%) who were exposed early to infliximab. 67% of pregnancies resulted in a live birth, 14% spontaneous miscarriages, and 19% therapeutic miscarriages, which was comparable to pregnancy outcomes in CD without infliximab treatment (Katz et al., 2004). Similarly, Schnitzler et al. found that overall pregnancy outcomes, except for abortion, with infliximab and adalimumab treatment at conception or during pregnancy were not different from pregnancy outcomes before anti-TNFα therapy. The same was found for patients exposed for a long time before pregnancy, but the pregnancy outcomes were less favorable than those in pregnancies preceding IBD diagnosis (Schnitzler et al., 2011). Abortion rates were similar among all groups in this study; however, in another prospective study, the rate of miscarriages was higher in exposed mothers than in pre-conceptionally exposed or non-exposed women (Verstappen et al., 2011). Further data suggested that treatment with anti-TNFα drugs before or during pregnancy was not associated with poor global pregnancy outcomes based on the sum of obstetric and neonatal outcomes (Casanova et al., 2013). In addition, data from medical records concerning pregnant women exposed to immunosuppressive drugs included 56 womens with first trimester exposure to anti-TNFα who showed no significant increased risk in fetal adverse events or congenital malformations (Cooper et al., 2014). Similarly, outcomes after infliximab exposure were not different from non-exposure outcomes as indicated by data derived from the TREAT registry of CD patients (Lichtenstein et al., 2018). A recent publication from the extensive Janssen’s global safety surveillance database of infliximab exposed mothers revealed the outcomes of 1850 pregnancies. Spontaneous abortion, preterm labor, low birth weight rates were 12.1, 9.2, and 3.6% respectively, parallel to population rates. It provided valuable evidence for future recommendations about the continuation of infliximab late after the 30th week since they found no cumulative outcome difference between those exposed in the first and third trimesters (Geldhof et al., 2020).

#### Risk of Congenital Anomalies with Exposure to TNFα Inhibitors

TNFα has a role during embryo development; however, transfer of mAbs against TNFα at the beginning of organogenesis is trivial, leaving developmental impact unlikely.

Early observational data reported a reasonable safety profile. However, a causal relationship between TNFα inhibitors and the occurrence of VACTERL anomalies (V: vertebrae anomalies; A: anal anomalies; C: cardiac anomalies; T: tracheal problems; E: esophageal problems; R: renal defects; L: Limb defects) in a child born to a mother with psoriasis exposed to etanercept during pregnancy was reported (Carter et al., 2006). Consequently, scanning the FDA database from 1999 to 2005 of children born to mothers exposed to infliximab and etanercept revealed a surprisingly high number of congenital malformations, mostly cardiac with a high association of at least one of the anomalies of VACTREL group. However, when compared to a general population-based database of congenital anomalies (EUROCAT), there was no increase in VACTREL distribution among children exposed to TNFα blocker (Crijns et al., 2011).

A comparative prospective study of 492 pregnancies with SARD exposed mostly to adalimumab, infliximab, and etanercept at least in the first trimester and 1,532 non-exposed pregnancies revealed higher rates of major birth defects mostly cardiac anomalies even after adjustments to maternal differences. In addition, higher rates of preterm labor and low birth weights were reported in the exposed group. It was not clear whether these findings were related to drug exposure or insufficient disease control (Weber-Schoendorfer et al., 2015). Although not statistically significant, a higher risk was also reported in two other studies but with no increase in VACTERL incidence (Casanova et al., 2013; Bröms et al., 2016). Data of 154 womens from the OTIS registry found no increase in major or minor birth defects after *in-utero* exposure to adalimumab, including cases exposed throughout pregnancy (Burmester et al., 2017). Similar data were reported by a prospective study with exposure to infliximab, adalimumab, and etanercept (Diav-Citrin et al., 2014). The Janssen infliximab safety database has recently reported a 2% congenital anomaly rate which is not considered abnormal (Geldhof et al., 2020).

Although the etanercept structure preserves the Fc domain, lower deposition in the placenta and lower affinity to FcRn than infliximab and adalimumab were reported in *ex vivo* studies (Porter et al., 2016; Eliesen et al., 2020a). There was no evidence of poor pregnancy outcomes or major congenital anomalies in a large retrospective cohort from US health plan research database affiliated with Optum. The study had some limitations including the lack of data on the exact dose of the drug, timing, and total duration of administration (Carman et al., 2017).

A prospective Italian multi-center study endorsed the safety of anti-TNFα drugs given pre-conception or early in pregnancy for treating inflammatory arthritis (Bazzani et al., 2015). In addition, two consecutive meta-analyses in IBD patients found overall favorable pregnancy outcomes with no significant risk for miscarriages, preterm labor, low birth weight, or congenital anomalies (Narula et al., 2014; Shihab et al., 2016). Later, pooled data from a systematic literature review leading to the 2016 European League Against Rheumatism (EULAR) recommendations for use of antirheumatic drugs before and during pregnancy and lactation reported no increase in the frequency of miscarriages or congenital anomalies with the use of anti-TNFα during gestation (Götestam Skorpen et al., 2016).

The safety of certolizumab offers an alternative to rule out the uncertainties related to other anti-TNFα drugs. Unlike other drugs, an experimental study found no binding affinity between certolizumab and placental FcRn *in vitro*, clearly due to the absence of the Fc portion, and slight or no placental transfer of certolizumab in *ex vivo* model (Porter et al., 2016). In a case series of 13 womens with RA and SpA treated with certolizumab throughout pregnancy, the drug was not detected or detected at low levels in the cord blood (Förger et al., 2016). These results were in accordance with the previous study (Mahadevan et al., 2013). Using a highly sensitive assay for measuring certolizumab concentration, the CRIB study proved the lack of transfer of the drug from mothers exposed after the 30th gestational week to their children as the concentrations in cord blood were below measurement or trivial (Mariette et al., 2018).

#### International Guidelines

The EULAR recommendations considered the continuation of infliximab, adalimumab, and golimumab in the first part of pregnancy and certolizumab and etanercept till the end of pregnancy (Götestam Skorpen et al., 2016). In the same year, BSR-BHPR guidelines recommended safe continuation of infliximab till the 16th week, adalimumab and etanercept till the end of the second trimester, and certolizumab throughout pregnancy (Flint et al., 2016). The latest recommendations issued by the American College of Rheumatology (ACR) strongly considered the continuation of certolizumab at conception and during pregnancy. In the case of infliximab, golimumab, adalimumab, and etanercept, the ACR recommendations considered their continuation during first and second trimesters and discontinuation in the third trimester if the disease is well controlled. If the disease is active, the conditional continuation of these biological agents can be considered (Sammaritano et al., 2020).

### Abatacept

Fewer safety data are available regarding biologics other than anti-TNFα. Abatacept is a recombinant selective fusion protein that modulates a co-stimulatory signal T-cell activation (Blair and Deeks, 2017) and approved for the treatment of RA, JIA, and Psoriatic arthritis (PsA) (US Food and Drug Administration, 2017a).

Administration of high doses of abatacept to animal models during organogenesis was not associated with congenital malformations. Like other IgG mAbs, placental transfer of abatacept was described resulted in disturbances in the immune functions of juvenile rats (Kumar et al., 2015; Bristol-Myers Squibb, 2017). Lower drug levels are reported in foetal than in maternal serum and abatacept can be detected in breast milk (Pham et al., 2012; Saito et al., 2019b).

A combined retrospective/prospective analysis estimated spontaneous abortion rates in abatacept-exposed women as 25.8%, comparable to rates in the general population. Half of them were also exposed to methotrexate early in pregnancy and the rate of congenital anomalies was increased in comparison with that reported in the general population (8.1% vs. 3–5%) (Kumar et al., 2015). Limitation of records about disease activity during pregnancy and comorbidities make such data less reliable. Current guidelines recommend stoppage of abatacept at conception and during pregnancy (Flint et al., 2016; Götestam Skorpen et al., 2016; Sammaritano et al., 2020). A running registry for pregnancies under abatacept treatment is still collecting additional safety data (Bristol-Myers Squibb, 2019).

### Tocilizumab

Tocilizumab is another recombinant humanized IgG1 mAb that targets the receptors of the pleiotropic cytokine IL-6 (Nishimoto and Kishimoto, 2008). It is indicated for the treatment of RA unresponsive to DMARDs and/or anti-TNFα therapy, polyarticular and systemic JIA, and giant cell arteritis (US Food and Drug Administration, 2019b).

Tocilizumab safety was supported by animal studies showing normal fetal development, average postnatal development and satisfactory immunoglobulin production (Sakurai et al., 2012). Placental transfer and cord levels of tocilizumab are lower than those of anti-TNFα IgG1 and natural IgG (Saito et al., 2019a; Tada et al., 2019; Moriyama et al., 2020).

Outcomes of prospectively and retrospectively followed pregnancies from Roche safety database were published in 2016. Most of the patients were exposed pre-conceptionally or in the first trimester with only 17 patients being exposed during the second and third trimesters. Compared to population rates, higher incidence of preterm labor and low birth weight was reported although the limited data about disease activity after tocilizumab suspension hindered the explanation of the cause of these high rates. There was no increase in congenital birth defects. Despite the insufficient number of cases, pregnancies with exposure beyond the first trimester had very good outcomes (Hoeltzenbein et al., 2016). Conflicting results were published about rates of spontaneous abortions related to tocilizumab. While this database and a case series reported a high percentage of spontaneous abortions (Hoeltzenbein et al., 2016; Weber-Schoendorfer and Schaefer, 2016), data from a smaller Japanese safety study did not find an increase in abortion rates or birth defects (Nakajima et al., 2016). It is recommended, by the EULAR, BSR-BHPR and ACR guidelines, that tocilizumab should be stopped prior to conception and during pregnancy though harm to the fetus is unlikely with unintentional exposure.

### Anti-B Cell Therapies (Rituximab, Belimumab)

Rituximab is a chimeric anti-CD20 mAb approved for the treatment of anti-TNFα non-responsive RA, Granulomatosis with Polyangiitis (GPA) and Microscopic Polyangiitis (MPA) (US Food and Drug Administration, 2012) and is used off-label in some situations such as refractory immune thrombocytopenia and lupus nephritis. Rituximab represents an attractive option to abort severe disease flares in pregnancy especially in SLE and for maintaining remission if received before conception since it continues to have a B cell modulating effect for a long duration beyond its half-life (approximately 110 days) (Breedveld et al., 2007). Giving rituximab to monkeys during organogenesis resulted in low B-cell count in their newborns. It was also shown to cross the placenta like other IgG mAbs and it was previously categorized as category C (US Food and Drug Administration, 2012).

Human pregnancy data about its safety are scanty. In a systematic review by Das et al., including studies on different diseases treated with rituximab around conception, rates of abortions and preterm labor were 12% and 47%, respectively. However, considering the retrospective nature of the studies included in that review, it is difficult to evaluate for confounding factors such as other medications, comorbidities, and disease activity status (Das et al., 2018).

Even when rituximab was administered near conception with persistence of maternal drug levels for weeks after the last infusion, most of the neonates born to mothers treated with rituximab had no congenital anomalies explained by minimal IgG transfer early during organogenesis (Thurlings et al., 2010; Das et al., 2018). Rituximab was considered only for emergency use in life threatening SLE disease activity during pregnancy and should be stopped at pre-conception (Götestam Skorpen et al., 2016; Sammaritano et al., 2020). The BSR-BHPR recommendations specified that rituximab should be stopped 6 months pre-conception (Flint et al., 2016).

Another anti-B cell therapy, belimumab, which is a fully human IgG1 mAb directed against the soluble form of the BAFF was approved by the FDA in 2011 for use in adult autoantibody-positive moderately active SLE (US Food and Drug Administration, 2019c).

Belimumab intravenously administered throughout pregnancy in animal studies was well tolerated by mothers and fetuses with no noticeable immunological or developmental adverse events except for a low B-cell population, which is expected (US Food and Drug Administration, 2019c). Although cumulative reports show some births with congenital anomalies, the data are insufficient to relate their occurrence to belimumab (Danve et al., 2014; Kumthekar et al., 2017; GlaxoSmithKline, 2020).

A registry is kept by GlaxoSmithKline to evaluate pregnancies and children born under belimumab exposure (GlaxoSmithKline, 2020). It would be important to confirm that anti-B cell therapy is a safe treatment option for SLE in pregnancy and breastfeeding since the disease has a serious potential for flaring up during pregnancy and postpartum period.

Recently, the ACR recommendations conditionally allowed the continuation of belimumab during conception which is a step forward from the earlier EULAR and BSR-BHPR recommendations that considered stopping it before conception. However, both still recommend against its continuation once pregnancy is confirmed (Flint et al., 2016; Götestam Skorpen et al., 2016; Sammaritano et al., 2020).

### IL-17 Inhibitors

Secukinumab followed by ixekizumab were the two FDA-approved IL-17 inhibitors for the treatment of moderate-to-severe plaque psoriasis, psoriathic arthritis (PsA), and ankylosing spondylitis (AS). They interfere with the pro-inflammatory activity of IL-17 which has been recently considered a key pathogenic player (Koenders and van den Berg, 2016; Chandran et al., 2020; Genovese et al., 2020). Secukinumab is a recombinant human monoclonal IgG1/κ antibody while ixekizumab is a humanized IgG4 mAb. Both can block the IL-17/IL-17R interaction through selective IL-17A neutralization (Frieder et al., 2018). Like other IgG molecules, secukinumab transfer starts after the 17th week and moreover throughout pregnancy (Saji et al., 1999; Simister, 2003).

Whereas nearly all clinical trials consider gestation as exclusion criteria, pregnancy outcomes using these monoclonal antibodies is remains uncertain. According to Novartis global safety database about outcomes of pregnancies mostly exposed to secukinumab at conception before stopping the drug in pregnancy, secukinumab was not related to abnormal rates of miscarriage or congenital abnormalities among the 292 study participants (238 maternal exposure) (Porter et al., 2017; Warren et al., 2018; Puchner et al., 2019). There are no accessible data about exposure throughout pregnancy. Accordingly, it is still recommended against its use in pregnancy by the European Medicines Agency (Warren et al., 2018; European Medicines Agency, 2019).

Studies on animal models found no embryo-fetal toxicity or teratogenic effects of secukinumab or ixekizumab although an increase in neonatal losses was reported in monkeys after exposure to ixekizumab at the 20th week of gestation (Clarke et al., 2015; Novartis Pharmaceuticals Corp, 2020). Of the two IL-17 inhibitors, secukinumab was mentioned only in the relevant ACR guidelines that recommended its discontinuation before conception and during pregnancy (Sammaritano et al., 2020).

### IL-12/IL-23 Pathway Blocker

Ustekinumab is a human IgG1/κ monoclonal antibody that blocks the p40 subunit of IL-12 and IL-23 cytokines (Benson et al., 2011). Ustekinumab has been introduced as an FDA approved bDMARD in the treatment of moderate to severe plaque psoriasis, active PsA, and IBD (US Food and Drug Administration, 2019a). Animal studies have reported no adverse effects on neonatal development during ustekinumab administration (Lund and Thomsen, 2017; US Food and Drug Administration, 2019a). Up to 2017, a total of 12 pregnancies in which ustekinumab was used has been reported by Venturin et al. Two out of 12 pregnancies have been aborted in the second and eighth weeks. However, the rest of the pregnancies were delivered uneventfully, and the babies were born without any anomalies (Venturin et al., 2017). According to a report on seven patients with psoriasis who were under ustekinumab treatment, 10 pregnancies resulted in eight healthy infants with no malformation (Watson et al., 2019). Maternal-fetal transfer of ustekinumab is assumed to be similar to other IgG1 mAbs. However, no new safety information was recorded by the most recent clinical reports. Therefore, they recommended that the final application be discontinued 8–12 weeks prior to parturition (Gisbert and Chaparro, 2020). According to both the EULAR and ACR statements, ustekinumab should be stopped before attempting conception and during pregnancy (Götestam Skorpen et al., 2016; Sammaritano et al., 2020).

### IL-1 Inhibitors

Inhibitors of IL-1 have been recently employed in the treatment of systemic autoinflammatory syndromes. Experience with therapeutic agents like anakinra, canakinumab, and rilonacept is limited. Anakinra is a recombinant protein that blocks IL1 receptor. Canakinumab is a full mAb while rilonacept is a fusion protein including the IL-1 binding motifs of IL-1 receptors coupled to the Fc domain of human IgG1. Few publications exist regarding their safety in gestation. Anakinra and canakinumab were well tolerated and pregnancy outcomes in small studies were satisfactory including cases of paternal and late pregnancy exposures (Chang et al., 2014; Youngstein et al., 2017; Venhoff et al., 2018).

It is noteworthy that oligohydramnios was previously reported, and renal agenesis was observed in two of anakinra exposed children which is worrisome considering the small number of the published cases (Chang et al., 2014; Youngstein et al., 2017). For the time being, anakinra seems to be safer to be prescribed in pregnancy than canakinumab with more evidence published and shorter half-life (Youngstein et al., 2017). The official recommendations still indicate that anakinra should be stopped before conception (Flint et al., 2016; Götestam Skorpen et al., 2016; Sammaritano et al., 2020).

Rilonacept studies in humans are lacking but animal pregnancy studies have raised concern about a relation between the drug and skeletal anomalies and fetal deaths (US Food and Drug Administration, 2008).

### Eculizumab

Eculizumab, the humanized anti-C5a antibody, was engineered as a unique IgG2/4κ formulation with weakened Fc functionality that limits its passage through the placental barrier and therefore theoretically renders it safer in pregnancy than other biologics (Hashira et al., 2000; Lozano et al., 2018; Sarno et al., 2019). It strongly binds C5 and interferes with its cleavage to the pro-inflammatory C5a and the terminal complement member C5b-9 (Schatz-Jakobsen et al., 2016). Eculizumab is approved by FDA in the treatment of atypical hemolytic uremic syndrome (aHUS), paroxysmal nocturnal hemoglobinuria (PNH), and generalized Myasthenia Gravis (gMG) (US Food and Drug Administration, 2017b).

It has also been shown to give good responses in refractory cases of catastrophic antiphospholipid syndrome (C-APS) and Hemolysis, Elevated Liver enzyme, and Low Platelet levels (HELLP) syndrome (Stefanovic, 2019). These all are serious conditions induced by complement activation that can be triggered by pregnancy, making the therapeutic use of eculizumab a possibility to be strongly considered. In the current literature, outcomes of only two triple antibody-positive APS patients treated with eculizumab during pregnancy were reported. Secondary thrombotic microangiopathy also complicated pregnancy in one of them. The two patients received eculizumab in the third trimester and delivered, prematurely by cesarean sections, healthy newborns (Gustavsen et al., 2017; Rovere-Querini et al., 2018).

Eculizumab was either not detected or detected at low ineffective titers in cord blood after administration in pregnancy including cases when mothers received it in late weeks. That is partially explained by its unique formulation and not by the strong binding between eculizumab and FcRn observed in*in vitro* studies. Poor pregnancy events were documented in some cases but attribution to the severity of the underlying condition as PNH or aHUS is more likely than to eculizumab itself (Kelly et al., 2010; Hallstensen et al., 2015; Miyasaka et al., 2016; Servais et al., 2016; Sarno et al., 2019). Eculizumab safety for use in pregnancy has not yet been addressed by the current rheumatology guidelines.

### Denosumab

Denosumab is a fully human IgG2 which is the least IgG subclass to be transferred through the feto-maternal interface. Its use for the treatment of pregnancy and lactation-associated osteoporosis, which is a relatively underreported condition with no identifiable treatment consensus, was addressed in some case reports with good results (Sánchez et al., 2016; Ijuin et al., 2017). However, it is contraindicated in pregnancy and lactation following discouraging animal studies which showed high still-birth and newborn mortality rates (Bussiere et al., 2013; Okamatsu et al., 2017).

A summary of the characteristics of the common drugs used in SARD including the risk category according to Food and Drug Administration (FDA) and Therapeutics Good Administration (TGA) is reported in Tables 1, 2.

**TABLE 1:** Common Biological Drugs in SARDs and their indications.

Biologic name (Trade name)	Target	Type	IgG subclass	Structure	Risk category		FDA approved indication	Common off label and investigatory indications in rheumatology
		Schmid and Neri (2019)			FDA	TGA Ngian et al. (2016)		
Infliximab (remicade)		Chimeric mAb	IgG1	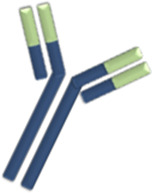	B	C	-AS	BD Hatemi et al. (2018)
							-RA	Sarcoidosis Loza et al. (2011).
							-Pediatric and adult CD and UC	Refractory OAPS Alijotas-Reig et al. (2019)
							-PsA and plaque Psoriasis	Pyoderma gangrenosum. Maverakis et al. (2020)
Adalimumab (humira)	TNFα	Fully human mAb	IgG1	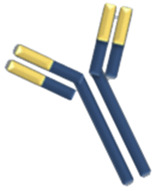	B	C	-As	
							-RA	
							-JIA	
							-Pediatric and adult CD and UC	
							-PsA and plaque psoriasis	
							-Uveitis	
Golimumab (simponi)		Fully human mAb	IgG1	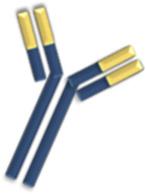	B	C	-As	
							-PsA	
							-RA -	
							UC	
Etanercept (enbrel)		Recombinant TNFα receptor added to Fcγ	IgG1	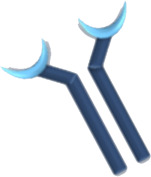	B	B2	-As	
							-RA	
							-Polyarticular JIA	
							-PsA and plaque psoriasis	
Certolizumab (cimzia)		Humanized fab fragment and PEG	—	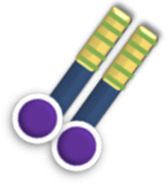	B	C	-RA	
							-As	
							-Non-radiographic axial SpA	
							-CD	
							-PsA	
Abatacept (orencia)	CD80	Extracellular region of CTLA-4 added to Fcγ	IgG1	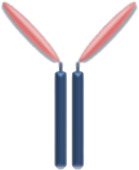	C	C	-RA	RA-ILD Fernández-Díaz et al. (2020)
	CD86						-JIA	
Rituximab (rituxan)	CD20	Chimeric mAb	IgG1	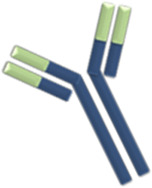	C	C	-RA	Refractory LN Stolyar et al. (2020)
								Refractory NPSLE Pamfil et al. (2015)
								Refractory IHA and thrombo-cytopenia Fanouriakis et al. (2019)
								ANCA associated vasculitis Yates et al. (2016)
								DM Oddis et al. (2013)
								SS Ramos-Casals et al. (2020)
								RA-ILD Dai et al. (2020)
								IgG4-RD Brito-Zerón et al. (2016)
Belimumab (benlysta)	BAFF	Fully human mAb	IgG1	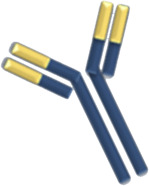	C	C	-Active, autoantibody positive SLE	Add-on therapy in LN Ward and Tektonidou (2020)
Secukinumab (cosentyx)		Humanized mAb	IgG1	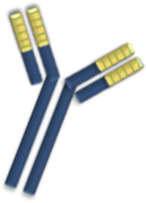	B	C	-As	NA
							-PsA and plaque psoriasis	
Ixekizumab (taltz)	IL-17	Fully human mAb	IgG4	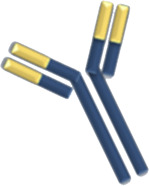	NA	C	-As	NA
							-PsA and plaque psoriasis	
Ustekinumab (stelara)	IL-12/IL-23	Fully human mAb	IgG1	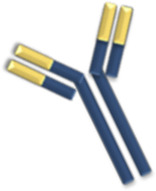	B	B1	-PsA	NA
							-CD and UC	
Tocilizumab (actemra)	C5	Humanized mAb	IgG1	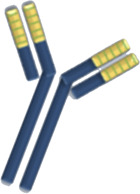	C	C	-RA	Castleman’s disease
							-GCA	PMR
							-Polyarticular and systemic JIA	Uveitis Rubbert-Roth et al. (2018)
							CRS	
Anakinra (kineret)	IL-1R	Recombinant non-glycosylated protein	—	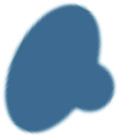	B	B1	-RA	AOSD
								BD
							-CAPS	FMF
								Systemic JIA
Canakinumab (ILARIS)	IL-1	Fully human mAb	IgG1	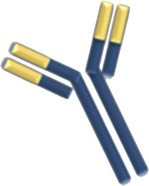	C	D/X	-FCAS	RA Vitale et al. (2016)
							-MWS	Gout So et al. (2018)
Rilonacept (arcalyst)	IL1	IL-1R binding motifs coupled to the Fc domain	IgG1	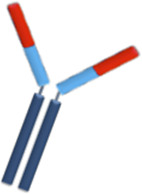	C	NA	-CAPS	Gout So et al. (2018)
							-FCAS	
							-MWS	
Eculizumab (soliris)	IL-6	Humanized mAb	IgG2/4	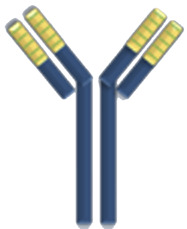	C	B2	-aHUS	HELLP
							-gMG	C-APS Stefanovic (2019)
							-PNH	
Denosumab (prolia/Xgeva)	RANKL	Fully human mAb	IgG2	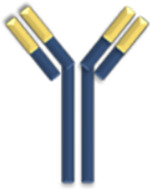	D	NA	Postmeno-pausal osteoporosis	PLO Sánchez et al. (2016)

NA, no available data; ACR, american college of rheumatology; aHUS, atypical hemolytic uremic syndrome; ANCA, anti-neutrophil cytoplasmic antibodies; AOSD, adult onset still’s disease; AS, ankylosing spondylitis; BAFF, B-cell activating factor; BD, behcet’s disease; C, complement; C-APS, catastrophic antiphospholipid syndrome; CAPS, cryopyrin-associated periodic syndromes; CD, crohn’s disease; CRS, cytokine release syndrome; DM, dermatomyositis; EULAR, european league against rheumatism; Fab, fragment antigen-binding; Fc, fragment crystallizable; FCAS, familial cold autoinflammatory syndrome; FDA, US food and drug administration; FMF, familial mediterranean fever; GCA, giant cell arteritis; gMG, generalized myasthenia gravis; IgG, immunoglobulin G; IHA, immune hemolytic anemia; IL, interleukin; ILD, interstitial lung disease; IgG4-RD, immunoglobutlin G4 related disease; JIA, juvenile idiopathic arthritis; LN, lupus nephritis; mAb, monoclonal antibody; MWS, muckle-wells syndrome; NPSLE, neuropsychiatric systemic lupus erythematosus; OAPS, obstetric anti-phospholipid syndrome; PEG, poly-ethylene glycol; PMR, poly-myalgia rheumatica; PNH, paroxysmal nocturnal hemoglobinuria; PLO, pregnancy and lactation associated osteoporosis; PsA, psoriatic arthritis; RA, rheumatoid arthritis; RANKL, receptor activator of nuclear factor kappa-Β ligand; SARD, systemic autoimmune rheumatic diseases; SS, sjögren syndrome; TGA, therapeutic goods administration; TNFα, tumor necrosis factor α; UC, ulcerative colitis.

**TABLE 2:** Risk categorization by FDA and TGA for use of drugs in pregnancy.

Category	FDA	TGA
A	Adequate and well-controlled studies have failed to demonstrate a risk to the fetus in the first trimester of pregnancy (and there is no evidence of risk in later trimesters).	Drugs which have been taken by a large number of pregnant women and women of childbearing age without any proven increase in the frequency of malformations or other direct or indirect harmful effects on the fetus having been observed.
B	Animal reproduction studies have failed to demonstrate a risk to the fetus and there are no adequate and well-controlled studies in pregnant women.	B1:
		Drugs which have been taken by only a limited number of pregnant women and women of childbearing age, without an increase in the frequency of malformation or other direct or indirect harmful effects on the human fetus having been observed.
		Studies in animals have not shown evidence of an increased occurrence of fetal damage.
		B2:
		Drugs which have been taken by only a limited number of pregnant women and women of childbearing age, without an increase in the frequency of malformation or other direct or indirect harmful effects on the human fetus having been observed.
		Studies in animals are inadequate or may be lacking, but available data show no evidence of an increased occurrence of fetal damage.
		B3:
		Drugs which have been taken by only a limited number of pregnant women and women of childbearing age, without an increase in the frequency of malformation or other direct or indirect harmful effects on the human fetus having been observed.
		Studies in animals have shown evidence of an increased occurrence of fetal damage, the significance of which is considered uncertain in humans.
**C**	Animal reproduction studies have shown an adverse effect on the fetus and there are no adequate and well-controlled studies in humans, but potential benefits may warrant use of the drug in pregnant women despite potential risks.	Drugs which, owing to their pharmacological effects, have caused or may be suspected of causing, harmful effects on the human fetus or neonate without causing malformations. These effects may be reversible.
**D**	Animal reproduction studies have shown an adverse effect on the fetus and there are no adequate and well-controlled studies in humans, but potential benefits may warrant use of the drug in pregnant women despite potential risks.	Drugs which have caused, are suspected to have caused or may be expected to cause, an increased incidence of human fetal malformations or irreversible damage. These drugs may also have adverse pharmacological effects.
**X**	Studies in animals or humans have demonstrated fetal abnormalities and/or there is positive evidence of human fetal risk based on adverse reaction data from investigational or marketing experience, and the risks involved in use of the drug in pregnant women clearly outweigh potential benefits.	Drugs which have such a high risk of causing permanent damage to the fetus that they should not be used in pregnancy or when there is a possibility of pregnancy.

## Biologic Drug-Induced Maternal and Infant Immunological Changes

Complex immunological changes naturally occur in pregnancy. Pregnant women are generally considered immunocompromized with more susceptibility to various infections (Mor and Cardenas, 2010). Administration of biological drugs surely induces additional variations in the maternal immune responses.

An insufficiently studied aspect is how much the drug level and efficacy are affected by pregnancy status. By measuring maternal infliximab and adalimumab levels each trimester in 25 pregnancies with IBD, an increase in infliximab with the progress of pregnancy was unexpectedly found. Adalimumab showed steady drug levels, although infliximab was discontinued earlier than adalimumab (Seow et al., 2017). Such findings were considered as a possible explanation for the higher cord blood levels of infliximab, which inversely correlated with the interval between the last dose and birth date, and the more delayed infliximab clearance from infant blood in former studies (Mahadevan et al., 2013; Julsgaard et al., 2016). Therefore, the authors concluded that monitoring anti-TNFα drug level, particularly in the second trimester, may offer a guide for third trimester dosing to ensure safety of the baby and protection to the mother from intrapartum or postpartum disease activity.

The 2014 meta-analysis by Narula et al. concluded that the benefit from anti-TNFα therapy overweighs any possible poor outcomes to the fetus or the mother (Narula et al., 2014). However, as their use is becoming more common, there are reports of a higher incidence of maternal infections that might be related to the use of anti-TNFα treatment in pregnancy. A study reported the records of twenty-five children exposed during gestation to infliximab/adalimumab; 80% of them were also breastfed till the median age of 6 months. Seven children had a mild decrease in immunoglobulin levels (IgA and IgG) without relevant clinical signs, while lymphocytic subpopulation counts were normal. Infections, mostly mild upper respiratory tract infections, were recorded in 80% of the babies. However, the reported infections do not seem to be therapy-related since they occurred late after the drug clearance (Bortlik et al., 2014). In the case series of women treated with certolizumab throughout pregnancy, maternal infections developed in three out of 13 included women; however, two of them were on concomitant low dose steroid therapy (Förger et al., 2016). A large cohort of French IBD patients was retrospectively investigated; 1,457 anti-TNFα (mostly infliximab and adalimumab) exposed patients were compared with 9,818 non exposed patients. Maternal infection rate in the first group was significantly higher than in non-exposed patients (Luu et al., 2018).

Another example of confusing maternal responses to biologic therapy during pregnancy is the need to increase doses and frequencies of eculizumab and increase the need for red blood cell transfusions in a subset of pregnant patients with PNH which might be explained by increased lysosomal metabolism of eculizumab and physiologic dilutional pregnancy changes (Kelly et al., 2015). Following treatment of a pregnant APS patient with eculizumab infusions, complement regained normal activity soon after infusions earlier than expected (Gustavsen et al., 2017). For the infant, the cord blood levels of various biologic drugs at birth display different values. Cord blood levels were high for infliximab and adalimumab (≈2.6 and 1.5% fetal to maternal ratio, respectively) (Mahadevan et al., 2013; Kanis et al., 2018), and not detected or negligible in the case of certolizumab, etanercept, and eculizumab (Murashima et al., 2009; Mariette et al., 2018). Most drugs with high levels at birth persisted in fetal blood for 4–9 months (Julsgaard et al., 2016).

Reversible change in immune cell counts is a well-recognized side effect particularly with the anti B-cell therapy. Neutropenia with serious skin infections was reported after treatment with infliximab in some reports (Guiddir et al., 2014). Apparently harmless reversible low infant total and B cell lymphocyte count without a significant rise in infection rates or abnormal vaccination course was reported after exposure to rituximab (Azim et al., 2010; Chakravarty et al., 2011; Das et al., 2018). A similar effect was seen in one case report with belimumab exposure (Bitter et al., 2018). These cellular effects were mostly transient and reverted to normal within months. Following eculizumab exposure, assessment of infant complement revealed normal activity (Hallstensen et al., 2015).

Since patients on anti-TNFα therapy display more susceptibility to mycobacterial infections due to interference with the IL-12/IFN-γ pathway, this pathway was investigated in newborns exposed to infliximab/adalimumab throughout pregnancy. Seven exposed and eight non-exposed children were followed for 12 months. Apart from one child who had recurrent infections after the 6th month of age, all showed no increase in infection rates with good developmental milestones. Leucocyte and lymphocyte counts were normal. T-regulatory cell values were lower than average in exposed children and defects in IL-12/IFN-γ pathway led to subnormal response to mycobacterial challenge. B-cell development and maturity are also thought to be affected by TNFα inhibition. Although this study showed that the B-cell population was shifted more to the naïve cells in exposed children, immunoglobulin levels were normal and antibody responses to diphtheria, tetanus, and pneumococcal vaccines were average (Esteve-Solé et al., 2017).

## Breastfeeding

The risk of transfer of biologics in breast milk is generally lower than that of placental transfer. Unlike IgA, overall secretion of IgG is scarce in breast milk. The preferential secretion of IgG subclasses is different from the placental one in which IgG3 and IgG4 are secreted more than IgG1 (Gasparoni et al., 1992). Transfer of the Fab fragment is even much lower than full IgG (Nesbitt et al., 2006). Moreover, mAb oral bioavailability is limited due to their large molecular size and the fact that degradation by proteolytic enzymes (Zelikin et al., 2016; Ryman and Meibohm, 2017). Nevertheless, FcRn are expressed at the intestinal mucosal surface of the newborn and can mediate the transfer of antibodies which escape proteolysis (Clowse et al., 2017; Pyzik et al., 2019).

Following these concepts, breast milk levels of almost all anti-TNFα and other mAb biologics are either very low or undetectable and mostly not be delivered to the nursing child’s circulation (Murashima et al., 2009; Ben-Horin et al., 2011; Clowse et al., 2017; Matro et al., 2018). Nevertheless, the reassurance of physicians for the use in clinical practice needs more studies reporting actual outcomes with breastfeeding.

The best evidence available is for certolizumab. It was not detected in the breast milk of nursing mothers following treatment with certolizumab (Mahadevan et al., 2013; Förger et al., 2016). A post-marketing multicenter study confirmed minimal or no excretion of certolizumab or PEG in breast milk and negligible relative infant dose of certolizumab (Clowse et al., 2017). In parallel, insignificant amounts of etanercept were detected in breast milk following treatment during breastfeeding and the drug was not detected in infant circulation (Murashima et al., 2009; Berthelsen et al., 2010; Keeling and Wolbink, 2010). Breast milk levels of other TNFα blockers and rituximab follow the same pattern as etanercept with low levels excreted in breast milk (Matro et al., 2018). The 2020 ACR guidelines considered all the TNFα inhibitors and rituximab compatible with breastfeeding with a strong recommendation although based on a low level of evidence. No data were available to support recommendations for its use during lactation by the EULAR and the BSR-BHPR guidelines. It is advised against nursing for 6 months after exposure to rituximab by FDA (US Food and Drug Administration, 2018).

There are no or very few data regarding other IgG1 mAbs (belimumab, tocilizumab, secukinumab, ustekinumab) and anakinra. They are conditionally recommended for use in pregnancy putting into consideration patient benefits vs. risks to the child (Sammaritano et al., 2020). Breast milk levels of belimumab were recently reported to be low at 1/200 to 1/500 of maternal values (Saito et al., 2020). Anakinra is profusely excreted in breast milk but infant drug levels have not yet been clearly measured. Since IL1 receptor antagonists are naturally present in breast milk, it was presumed that anakinra would not have a detrimental effect on the infant. That was confirmed by several studies that showed average developmental progress in children breastfed by mothers receiving anakinra (Chang et al., 2014; Youngstein et al., 2017; Smith and Chambers, 2018). Similarly, the IgG2/4κ eculizumab was not detected in breast milk samples and no developmental problems were published (Kelly et al., 2015; Miyasaka et al., 2016).

Table 3 summarizes the guidelines for using biological drugs during pregnancy, lactation, and for male partners according to ACR, BSR/BHPR and EULAR committees.

**TABLE 3:** Guidelines for continuation of biological drugs in pregnancy, lactation and male partners.

Biologic name	Outcomes of pregnancy animal studies^a^	ACR	EULAR	BSR/BHPR
		Pregnancy/Breastfeeding/Paternal Sammaritano et al. (2020)	Pregnancy/Breastfeeding/Paternal Götestam Skorpen et al. (2016)	Pregnancy/Breastfeeding/Paternal Flint et al. (2016)
Infliximab	Infliximab analogue did not result in fetal malformations or developmental effects in mice offspring.	Conditional^b^/Continue/Continue	Stop at week 20/Continue/-	Stop at week 16/Continue/Continue
Adalimumab	Exposing cynomolgus monkeys to doses higher than the MHRD did not result in fetal malformations or harms.		Stop at week 20/Continue/-	Conditional^b^/Continue/Continue
Golimumab	No neonatal or postnatal developmental harm with pregnancy and breastfeeding in monkeys.		Conditional^b^/NA/-	NA/NA/NA
Etanercept	No evidence of mal-formations in rats and rabbits exposed to etanercept during embryogenesis. Normal post-natal development of rats exposed to doses 48 times the human dose.		Continue/Continue/-	Conditional^b^/Continue/Continue
Certolizumab	Studies in rats using antimurine analogue resulted in no fetal harms or malformations	Continue/Continue/Continue	Continue/Continue/-	Continue/Continue/Continue
Abatacept	In-utero and juvenile exposure of rats to doses 11 times the MHRD resulted in disturbance in fetal immune response.	Discontinue/Conditional/NA	Discontinue^c^/Discontinue^c^/-	Discontinue/NA/NA
Rituximab	Animal exposure showed no teratogenic effect but decrease in B-cell lymphoid tissue and reversible decline in B-cell population.	Conditional^d^/Conditional^d^/Continue	Discontinue^c^/Discontinue^c^/-	Discontinue 6 mths pre-conception/NA/Continue
Belimumab	Exposure of monkeys throughout pregnancies to high doses resulted in no fetal malformations. Fetal decrease in peripheral and lymphoid tissue B-cell population, increase in total IgG and decrease in IgM were reported. All changes recovered within the first year of life.	Discontinue/Conditional^e^/NA	Discontinue^c^/Discontinue^c^/-	Discontinue/NA/NA
Secukinumab	No fetal toxicity or malformations on exposure to high doses.	Discontinue/Conditional^e^/NA	—	—
	Secukinumab analogue did not lead to abnormal morphological or immunological effects In a pre- and post-natal developmental study.			
Ixekizumab	No fetal toxicity or malformations was observed on exposure to high doses	—	—	—
	In pre and post-natal developmental studies, no abnormal morphological or immunological effects. However, early neonatal deaths were reported, unlikely due to ixekizumab.			
Ustekinumab	In embryofetal and developmental studies, exposure >100 times higher than the human SC exposure resulted in no fetal toxicities, malformations, developmental, morphological or immunological effects. Unexplained 2 neonatal deaths were reported.	Discontinue/Conditional^e^/NA	Discontinue^c^/Discontinue^c^/-	—
Tocilizumab	No fetal toxicity, malformations, developmental or immunological abnormalities were reported.	Discontinue/Conditional^e^/NA	Discontinue^c^/Discontinue^c^/-	Discontinue 3 mths pre-conception/NA/NA
	Increase in rate of abortions was reported in monkeys at doses 1.25 higher than the MRHD.			
Anakinra	Studies on rabbits and mice at doses 25 times the MRHD resulted in no fetal harm.	Discontinue/Conditional^e^/Conditional	Discontinue^c^/Discontinue^c^/-	Discontinue/NA/NA
Canakinumab	Studies on monkeys at doses 11 times the MRHD showed no malformations. Rate of skeletal developmental delay due to incomplete ossification was increased.	—	—	—
Rilonacept	Study on monkeys reported rib and vertebral abnormalities. There was also increase in rate of stillbirths and neonatal deaths.	—	—	—
Eculizumab	Exposure of mice to doses up to 8 times the MRHD early in pregnancy resulted in no abnormality. Exposure during organogenesis resulted in 2/230 neonates with retinal dysplasia. Exposure from implantation to weaning was associated with higher death rate among pups. All live births showed normal development.	—	—	—
Denosumab	In monkeys, high still-birth and newborn mortality rates due to intrinsic fetal defects such as increased bone mass, insufficient hematopoiesis, hypoglycemia, and hypocalcemia.	—	—	—
	Abnormal bone growth with decreased strength, absence of lymph-node groups, dental dysplasia and reduced neonatal growth were reported in the offspring.			

^a^ Animal experimental data were extracted from FDA labes of each drug.

^b^ Stop before third trimester.

^c^ Discontinue except if no pregnancy compatible alternative to control activity.

^d^ Consider use in organ/life threatening conditions.

^e^ Limited available data but expected minimal transfer in breast milk.

NA: No available recommendation due to limited published data. — Not mentioned in the guidelines. MHRD, maximum human recommended dose.

The data from animal studies are also summarized in Table 3.

## Vaccine Responses and Long-Term Outcomes

More informative long-term data regarding outcomes of children after exposure to biological drugs *in-utero* or during nursing are needed. In addition, precise assessment of responses to vaccines, especially for the first-year doses, is definitely needed to ensure successful immunization after inactivated vaccines and exclude the risk of serious side effects with live-attenuated vaccines. Anticipated persistence of mAb biologics in infant blood and B-lymphocyte depletion following exposure to anti B-cell therapy requires more research to determine the time of clearance of different biologics and proper planning of an effective and safe vaccination schedule. Reports about responses to inactivated vaccines are more available than live-attenuated since it is a common practice to delay live-attenuated vaccines till after the first year.

Although data from drug registries and observational studies report minimal or no side effects in infants exposed to infliximab *in-utero*, early reports revealed a serious disarray in infants` immune responses to Bacille Calmette–Guérin (BCG) vaccines (Cheent et al., 2010).

A study showed that, postnatally, infliximab was cleared from infant blood over a longer duration than adalimumab (mean 7.3 vs. 4 months), raising concerns about infant`s response to live-attenuated vaccines, particularly BCG, which is still obligatory in many countries (Julsgaard et al., 2016). In this study, authors found that 4/80 (5%) of infants suffered bacterial infections and 16/80 (20%) had viral infections, but most of them were exposed to anti-TNFα *in-utero* and also to thiopurines.

During a 2-years follow-up period, 34 children exposed *in-utero* to anti-TNFα agents (8 after the first trimester) showed no difference in growth and developmental parameters nor in the incidence of congenital malformations in comparison to controls. Post-vaccination responses and adverse events were also similar except for one etanercept exposed child who contracted chickenpox infection after insufficient response to the relevant vaccine (Dall'ara et al., 2016). The World Congress of Gastroenterology statements in 2011 recommended giving special attention to the timing of biological therapy during pregnancy and postponing delivery of live-attenuated vaccines to exposed children until the drug is no longer detected in their circulation (Mahadevan et al., 2011).

According to Bortlik et al., serologic antibody responses to first year scheduled inactivated vaccines were found satisfactory except for *Hemophilus influenza* B (HiB) vaccine which produced below threshold protective antibody levels in 6/15 examined children (Bortlik et al., 2014). In a recent evaluation of the long-term impact of *in-utero* exposure to anti-TNFα therapy, Duricova et al. followed 72 exposed children older than 12 months of age and compared them with 69 non-exposed children for a duration of follow up that ranged from 3 to 4 years. Most of the mothers had CD and were treated with infliximab, which was used until 17–30 weeks of gestation. There was no significant variation between groups of children regarding growth, psychomotor development, allergy status, and infection rate during the first year of life and the period of follow-up (Duricova et al., 2019). Vaccines caused more adverse events in the exposed group, but that difference was not statistically significant, and none of the adverse events was critical (23 vs. 11.6% *p* = 0.06). Of the children who received scheduled vaccines, serologic response was adequate in >95% of exposed children except for HiB and mumps vaccines, for which fewer children in exposed and control groups had an adequate serologic response (65.3 vs 12.5%) and (75.7 vs. 81.3%) of tested children (Duricova et al., 2019). In another study, children exposed to biologics (including anti-TNFα and ustekinumab) during breastfeeding in the first year of life showed average developmental milestones and infection rates similar to controls (Matro et al., 2018). Another set of extracted data from the Pregnancy in Inflammatory Bowel Disease and Neonatal Outcomes registry (PIANO) registry was published in 2018 reporting infant serologic responses to HiB and tetanus vaccines after antenatal exposure to biologics including anti-TNFα and ustekinumab. The percentage of infants who had protective serological titers after HiB and tetanus vaccines did not differ between the exposed and non-exposed children, but the comparison group was small and included infants exposed to other immunosuppressive and immunomodulatory agents. Moreover, the response to vaccines was not related to the different titers of the biologics in cord blood at birth. Most of the mothers avoided the live-attenuated Rotavirus vaccine based on medical advice. Sequelae, including diarrhea and fever, were reported in 17% (7/40) of infants receiving the Rotavirus vaccine after exposure to anti-TNFα drugs (Beaulieu et al., 2018).

In an effort to determine the ability to achieve an adequate serological response after receiving an inactivated vaccine, de Lima et al. measured anti-hepatitis B antibodies one year after vaccination and proved that effective vaccination could be achieved in anti-TNFα exposed children similar to non-exposed children. Growth and developmental outcomes were also similar between the two groups at one year of age (de Lima et al., 2018). Recently a Korean study examined 12 children who were exposed to infliximab/adalimumab *in-utero* with a mean age of 28.3 ± 16.6 months. All showed average growth and development: 33% needed a booster dose after hepatitis B virus (HBV) vaccination for seroconversion which was similar to other non-exposed Korean children. Seven out of 12 children received live-attenuated vaccines before 6 months of age without complications (Lee et al., 2019).

Following the early discouraging results, BCG vaccine was given at different ages during the first year of life in various observational studies with generally few adverse events, but the relationship to the timing of the last dose of biologic therapy was sometimes poorly reported (Bortlik et al., 2014; Luu et al., 2018). Proper timing for BCG vaccination was investigated by retrospectively analyzing records of offspring of 74 women treated by anti-TNFα drugs during pregnancy, including those who received third trimester doses. Adverse events were very few and self-limited in infants who received BCG vaccine at a range of 0.25–11 months and totally absent in infants receiving it at or after the 6th month of age. The authors eventually recommended 6 months after birth as the optimal timing for delivering the BCG vaccine and the same recommendation may be applied to other live-attenuated vaccines (Park et al., 2020).

Few more results about some biologic drugs other than anti-TNFα are continuously published showing supporting evidence of long-term safety. Sixteen children were followed up to 1 year (6–48 weeks) after abatacept exposure in one study. No abnormal increase in infection rate or immunodeficiencies was observed (Kumar et al., 2015). Follow up of breastfed children exposed to anakinra since fetal stage and up to 10 years postnatal in some cases was mostly uneventful with no serious infections or developmental delays (Youngstein et al., 2017; Smith and Chambers, 2018). Response to vaccination after rituximab has been discussed in case reports. All showed adequate antibody responses to vaccines, including Tetanus, Diphtheria, HBV, HiB, MMR, and polio (Decker et al., 2006; Friedrichs et al., 2006; Mandal et al., 2014; Ling and Koren, 2016), except in one case report with an insufficient response after Diphtheria (Kimby et al., 2004).

## Indirect Exposure to Biologic Therapy via the Male Partner

A limited number of studies addressed paternal exposure to biological drugs, infertility, and pregnancy outcomes in the current literature. Cohort studies have reported no relevant difference between peri-conceptional paternal exposure to different anti-TNFα and undesirable pregnancy outcomes or congenital malformations with rates comparable to population rates (Viktil et al., 2012; Wallenius et al., 2015; Larsen et al., 2016).

The majority of papers reported no impact of TNFα inhibitors on male fertility (Saougou et al., 2013; Micu et al., 2014; Mouyis et al., 2019). Only a few reported decreased spermatozoa number or questionable abnormality of motility and morphology in relation to infliximab (Mahadevan et al., 2005; Montagna et al., 2005).

With other drugs, outcomes of 10, 54, and 13 pregnancies after paternal exposure to abatacept, secukinumab, and tocilizumab were satisfactory with the majority achieving healthy live births free of congenital anomalies (Kumar et al., 2015; Hoeltzenbein et al., 2016; Warren et al., 2018).

Followed pregnancies after rituximab treatment in the male partner were few. Only 9 of 22 pregnancies could be followed in one study with seven live births, four premature infants, and two spontaneous miscarriages (Chakravarty et al., 2011).

Unlike the EULAR recommendations in 2016 (Götestam Skorpen et al., 2016), the BSR-BHPR and the recent ACR guidelines devoted statements for prescribing antirheumatic drugs in male partners with SARD planning to conceive a pregnancy. Both guidelines recommended the continuation of infliximab, adalimumab, etanercept, and rituximab in men attempting to conceive. The BSR-BHPR did not make recommendations for certolizumab, and anakinra use in breastfeeding due to lack of supporting data, while the ACR guidelines strongly recommended certolizumab and conditionally recommended anakinra continuation (Flint et al., 2016; Sammaritano et al., 2020). Relevant recommendations for other biologics were not made due to limited data.

Table 4 summarizes the data on the transfer of the different biologicals and their effect on infants.

**TABLE 4:** Characteristics of transfer of different biologics and their effect on the infants.

Drug	Placental transfer	Reported infant level at birth	Persistence in child blood after birth.	Transfer through breastfeeding	Reported child immunological changes	Relevant outcomes within the first year of life and vaccination responses^a^
Infliximab	Yes Julsgaard et al. (2016)	High, up to 2.6 infant to maternal ratio Kanis et al. (2018)	Persist for mean duration of 7.3 months Julsgaard et al. (2016)	Yes, at very low levels Matro et al. (2018)	In some cases, neutropenia, serious skin, bacterial and viral infections were reported Guiddir et al. (2014).	Overall, no serious adverse events Beaulieu et al. (2018).
Adalimumab	Yes Julsgaard et al. (2016)	High, up to 1.5 infant to maternal ratio Mahadevan et al. (2013)	Persists for mean duration of 4 months Julsgaard et al. (2016)	Yes, at very low levels Matro et al. (2018)	Altered T- and B-regulatory compartment, increased eosinophil counts in cord blood Esteve-Solé et al. (2017)	Abnormal infant’s’ immune response to BCG vaccine in one case of infliximab exposure Cheent et al. (2010).
Golimumab	Expected^b^ Martin et al. (2007)	NA	Persists until 6 months in animal studies. Martin et al. (2007)	Very low Matro et al. (2018)	No reported immunological changes Martin et al. (2007)	Adequate serological response except for hemophilus influenza B and mumps vaccines after adalimumab exposure Duricova et al. (2019).
Etanercept	Low Berthelsen et al. (2010)	Cord blood level is negligible. Infant to maternal ratio is 0.04 Berthelsen et al. (2010)	-In one case, persisted for 3 months. Berthelsen et al. (2010)	Insignificant Murashima et al. (2009)	No reported immunological changes.	After rota virus vaccine, 17% of vaccinated children had diarrhea and fever Beaulieu et al. (2018).
Certolizumab	Almost no placental transfer Mariette et al. (2018)	Not detected/negligible infant to maternal ratio is 0 Mariette et al. (2018)	No Mariette et al. (2018)	Minimal/not detected Clowse et al. (2017)	No reported immunological changes	No reported abnormal growth or developmental changes.
Abatacept	Expected^b^ Bristol-Myers Squibb (2017)	NA	NA	Very low Saito et al. (2019b)	No reported immunological changes	No adverse events were reported. No reported abnormal growth or developmental changes Saito et al. (2019b).
Rituximab	Yes (US Food and Drug Administ-ration, 2018)	Higher than maternal level Klink et al. (2008)	NA	Very low Bragnes et al. (2017)	Reversible decrease in infant total and B-cell lymphocytic counts Das et al. (2018)	Adequate antibody response to vaccines except in one case report with insufficient response after diphtheria Diphtheria. No reported abnormal growth or developmental changes. Kimby et al. (2004).
			The biological effect persists up to 6 months due to its long half-life Østensen (2017)			
Belimumab	Yes GlaxoSmithKline, (2020)	NA	NA	Very low Saito et al. (2020)	Reversible decrease in infant total and B-cell lymphocytic counts Bitter et al. (2018)	Adequate responses with no adverse events. No reported abnormal growth or developmental changes Bitter et al. (2018); Saito et al. (2020).
Secukinumab	Expected^b^ after 17th week Warren et al. (2018)	NA	NA	NA	NA	NA
Ixekizumab	Yes Clarke et al. (2015)	NA	NA	NA	NA	NA
Ustekinumab	Expected^b^ (Gisbert and Chaparro (2020)	High, infant to maternal ratio up to 1.4–2:1 Mahadevan et al. (2017); Rowan et al. (2018)	NA	Very low Matro et al. (2018)	No reported immunological changes	NA on vaccine response No reported abnormal growth or developmental changes.
Tocilizumab	Yes, lower than other IgG molecules Moriyama et al. (2020)	Low drug levels were detected in cord blood Saito et al. (2019a).	Persists until 1–2 months Saito et al. (2019a)	Very low Saito et al. (2019a)	No reported immunological changes or severe infections.	No adverse events were reported. No reported abnormal growth or developmental changes Saito et al. (2019a).
Anakinra	NA	NA	-NA	High Buescher and Malinowska, (1996).	No reported immunological changes or severe infections.	No adverse events were reported Duman et al. (2019). No reported abnormal growth or developmental changes up to 10 years.
Eculizumab	Yes, lower than IgG1 Eliesen et al. (2020b)	Not detected/very low cord blood level Sarno et al. (2019)	-NA	Undetectable Sarris et al. (2012)	No reported immunological changes normal infant complement activity	No adverse events were reported. No reported abnormal growth or developmental changes.
Denosumab	Yes Bussiere et al. (2013)	Detected in animal studies. Bussiere et al. (2013)	NA	Yes, likely low levels in breast milk in animal studies. Bussiere et al. (2013)	NA	NA

^a^ A lot of the vaccination reports did not include data about live-attenuated vaccines since they are usually delayed after 6 months of age.

^b^ Expected based on the transfer of IgG1 molecules through placenta.

NA: No available data.

## Discussion

Ensuring safety of biological therapies during preconception, pregnancy, and breastfeeding offers an important treatment option in disorders in which disease control is crucial. Several reports and observational studies are reassuring especially in dealing with anti-TNFα therapies. Biologics may offer a safer choice than non-biologic drugs in regard to neonatal infections and congenital anomalies. Heterogeneity in study design, disease populations, target biologics studied, the timing of exposure to biologics, outcome measures, inadequate comparison groups and difficulty in adjustment for confounders such as disease activity do not provide very strong evidence for recommendations.

The largest experience is from IBD cases with exposure to anti-TNFα in pregnancy. TNFα inhibitors seem to be safe and effective without a significant maternal or fetal risk despite previous worries about the risk of an increase in congenital malformation. Except for certolizumab, a PEGylated monovalent Fab fragment, all other available anti-TNFα drugs cross the placental and can be detected in fetal blood. Eculizumab is another biologic which recently showed limited placental passage owing to the unique engineered structure of IgG2/4. A comparable low placental transfer was described for etanercept as well because of its molecular structure. While some unfavorable pregnancy outcomes like low birth weight and preterm labor were reported with the use of biologics in pregnancy, it is arguable that they may be a consequence of the autoimmune disease itself. Evidence about the safety of other biologics has not yet been sufficient. Exposure through fathers has shown safe outcomes.

Some studies have reported alterations in maternal and infant immune systems; however, the whole spectrum of these changes and their effects need to be further evaluated. First-year tracking of exposed children is comforting. Live-attenuated vaccines are still avoided in clinical practice at least for the first 6 months of life due to the anticipated persistence of some mAbs. Proper timing of biological dosing in pregnancy is yet to be more clearly determined. Longer follow-up periods are still needed to monitor for possible late effects like the risk of malignancy and delayed immunological effects in the exposed children. Assessment of available biosimilar drugs’ safety is also crucial since they provide more affordable therapy than biologic drugs. Only one paper published information about outcomes of a small number of pregnancies after peri-conceptional treatment by biosimilar infliximab with results not different from the original (Kolar et al., 2018).

Overall, the available data are encouraging. With the emergence of more studies every year, recommendations are stronger for considering treatment by certain biological drugs throughout gestation and breastfeeding.
